# Protocol for an Effectiveness-Implementation Hybrid Trial to Evaluate Scale up of an Evidence-Based Intervention Addressing Lifestyle Behaviours From the Start of Life: INFANT

**DOI:** 10.3389/fendo.2021.717468

**Published:** 2021-11-08

**Authors:** Rachel Laws, Penelope Love, Kylie D. Hesketh, Harriet Koorts, Elizabeth Denney-Wilson, Marj Moodie, Vicki Brown, Kok-Leong Ong, Jennifer Browne, Sarah Marshall, Sandrine Lioret, Liliana Orellana, Karen J. Campbell

**Affiliations:** ^1^Institute for Physical Activity and Nutrition, School of Exercise and Nutrition Science, Deakin University, Geelong, VIC, Australia; ^2^Sydney Nursing School, Faculty of Medicine and Health, University of Sydney and Sydney Local Health District, Sydney, NSW, Australia; ^3^Deakin Health Economics, Institute for Health Transformation, School of Health and Social Development, Deakin University, Geelong, VIC, Australia; ^4^La Trobe Analytics Lab, La Trobe University, Melbourne, VIC, Australia; ^5^Institute for Health Transformation, School of Health and Social Development, Deakin University, Geelong, VIC, Australia; ^6^Université de Paris, CRESS, INSERM, INRAE, Paris, France; ^7^Biostatistics Unit, Deakin University, Geelong, VIC, Australia

**Keywords:** child obesity prevention, infants (birth to 2 years), nutrition, physical activity, sedentary behavior, implementation science, obesity, scaling up

## Abstract

**Introduction:**

Promoting healthy eating and active play in early life is critical, however few interventions have been delivered or sustained at scale. The evaluation of interventions at scale is a crucial, yet under-researched aspect of modifying population-level health behaviours. INFANT is an evidence-based early childhood healthy lifestyle intervention that aims to improve parents’ knowledge and skills around promoting optimal energy balance-related behaviours that, in turn, influence children’s diet, activity and adiposity. It consists of: 1) Four group sessions delivered via first time parent groups across the first 12 months of life; 2) access to the My Baby Now app from birth to 18 months of age. This research aims to assess real-world implementation, effectiveness and cost-effectiveness of INFANT when delivered at scale across Victoria, Australia.

**Methods and Analysis:**

A hybrid type II implementation-effectiveness trial applying a mixed methods design will be conducted. INFANT will be implemented in collaboration with practice and policy partners including maternal and child health services, population health and Aboriginal health, targeting all local government areas (n=79) in Victoria, Australia. Evaluation is based on criteria from the ‘Outcomes for Implementation Research’ and ‘RE-AIM’ frameworks. Implementation outcomes will be assessed using descriptive quantitative surveys and qualitative interviews with those involved in implementation, and include intervention reach, organisational acceptability, adoption, appropriateness, cost, feasibility, penetration and sustainability. Process measures include organizational readiness, fidelity, and adaptation. Effectiveness outcomes will be assessed using a sample of INFANT participants and a non-randomized comparison group receiving usual care (1,500 infants in each group), recruited within the same communities. Eligible participants will be first time primary caregivers of an infant aged 0-3 months, owning a personal mobile phone and able to communicate in English. Effectiveness outcomes include infant lifestyle behaviours and BMIz at 12 and 18 months of age.

**Impact:**

This is the first known study to evaluate the scale up of an evidence based early childhood obesity prevention intervention under real world conditions. This study has the potential to provide generalisable implementation, effectiveness and cost-effectiveness evidence to inform the future scale up of public health interventions both in Australia and internationally.

**Clinical Trial Registration:**

Australian and New Zealand Clinical Trial Registry https://www.anzctr.org.au/, identifier ACTRN12620000670976.

## 1 Introduction

The prevention of obesity in early life is a key priority identified by the World Health Organization (1). The dietary and activity (energy-balance) behaviours associated with overweight and obesity begin in and track from early childhood (2). In Australia, a minority of children meet dietary, physical activity or sedentary behaviour guidelines (3). Children experiencing rapid weight gain during the first two years of life are also nearly four times more likely to become overweight or obese later in life (4). Globally, an estimated 41 million children aged under five are affected by overweight or obesity (5), including nearly one quarter of Australian children aged 2-4 years (3). Addressing energy balance behaviours in early life is therefore critical (6).

The prevalence of overweight and obesity, and the energy balance behaviours that predict them, are culturally, geographically and socioeconomically patterned (7–9) such that the most disadvantaged are disproportionately affected (8). It is estimated that obesity and dietary risks are each responsible for approximately 15% of the gap in disease burden between Aboriginal and Torres Strait Islander peoples and non-Indigenous Australians (10). Preventive interventions must therefore reach families across social, cultural and geographic divides. Most population-based interventions to improve children’s health behaviours in early life occur *via* settings, such as child care. These do not reach most children in their first year of life, where the risks for later overweight and obesity begin. At this time, the main influences on children’s health behaviours are the family and home environment (11) with a key avenue for reaching parents through Maternal and Child Health (MCH) services.

In Australia, routine universal MCH services are provided in all Australian states and territories. In Victoria, Australia, this service includes 10 free ‘key age and stage’ consultations with a MCH nurse (nine occurring between birth and two years of age) with a strong focus on promoting optimal child development and growth (12). More than 95% of Victorian parents attend these consultations between birth and four months, with over 80% still attending at 12 months (12). Routine preventive care services, such as those provided through MCH services, therefore offer a circumscribed opportunity to support parents in the establishment of healthy behaviours during infancy and early childhood.

The Infant feeding activity and Nutrition Trial (INFANT) (13) tested the efficacy of a group-based intervention delivered via established first time parents*’* groups by universal MCH services. The intervention aimed to improve parents*’* knowledge and skills around promoting healthy eating and active play, and in turn, healthy growth from the start of life (14). The intervention was underpinned by the principles of anticipatory guidance and social support and was first delivered in the trial by trained dietitians. Six 1.5-2hour face-to-face group sessions with parents and infants were delivered at 3 monthly intervals when the infant was approximately 3, 6, 9, 12, 15 and 18 months of age. The randomized controlled trial (RCT) showed INFANT achieved high acceptance, with 70% of participants attending ≥ four of six sessions over 15 months and 85% reporting high usefulness (13, 15). While the RCT did not impact standardised body mass index (BMIz) or physical activity (13), it did demonstrate positive child outcomes at 18 months for television viewing and dietary quality (16), with additional benefits for children of younger and less educated mothers (17). Additional positive outcomes were observed for mothers*’* own dietary patterns and feeding practices (15, 18). Importantly, despite no further intervention beyond 18 months of age, the impact of INFANT remained evident up to 5 years of age for several targeted child health behaviors but not adiposity (19, 20). It is important to note that as with most research trials, participants were predominantly well educated (54% had a university education or higher) and English speaking (94% spoke English as the main language at home). No data was collected on participation by Aboriginal and Torres Strait Islander families but it is likely they were under represented in the trial.

While evidence is accumulating in support of efficacious early life childhood obesity prevention interventions such as INFANT (21), few studies have examined the effectiveness of these interventions when implemented at scale, in a real-world context. Further, implementation evidence including information on reach (particularly amongst hard to reach groups), feasibility, acceptability, scalability, effectiveness and cost-effectiveness as well as sustainability are also lacking but vital to decision makers (22). This was reflected in a recent research prioritization exercise with child obesity prevention researchers, policy makers and practitioners who identified ‘implementation science’ and ‘how to integrate child obesity prevention into existing service structures’ as key priorities (23).

Interventions that can be integrated into existing delivery systems are more likely to be sustainable than resource intensive interventions (1). INFANT represents a low intensity intervention with the potential for wide scale integration into service delivery structures. In Victoria, Australia, INFANT is now promoted as a flagship state-wide intervention delivering on two priority areas within the Victorian Public Health and Well Being Plan (2019-23) (24) and is endorsed as an evidenced based program nationally by the Australian Institute of Family Studies. This provides an opportunity to evaluate the usefulness of implementation strategies and overall intervention effectiveness when delivering INFANT at scale in a real-world context.

## 2 Methods and Analysis

### 2.1 Study Aims and Objectives

This research aims to assess real-world implementation, effectiveness and cost-effectiveness of INFANT when delivered at scale across Victoria, Australia. It has two main objectives:

1) To assess reach, and system/organizational level barriers and enablers to the adoption, implementation and sustainability of INFANT when delivered at scale.
2) To assess effectiveness and cost-effectiveness of INFANT when delivered at scale.

### 2.2 INFANT Enhancements for Scale Up

**Figure 1:**
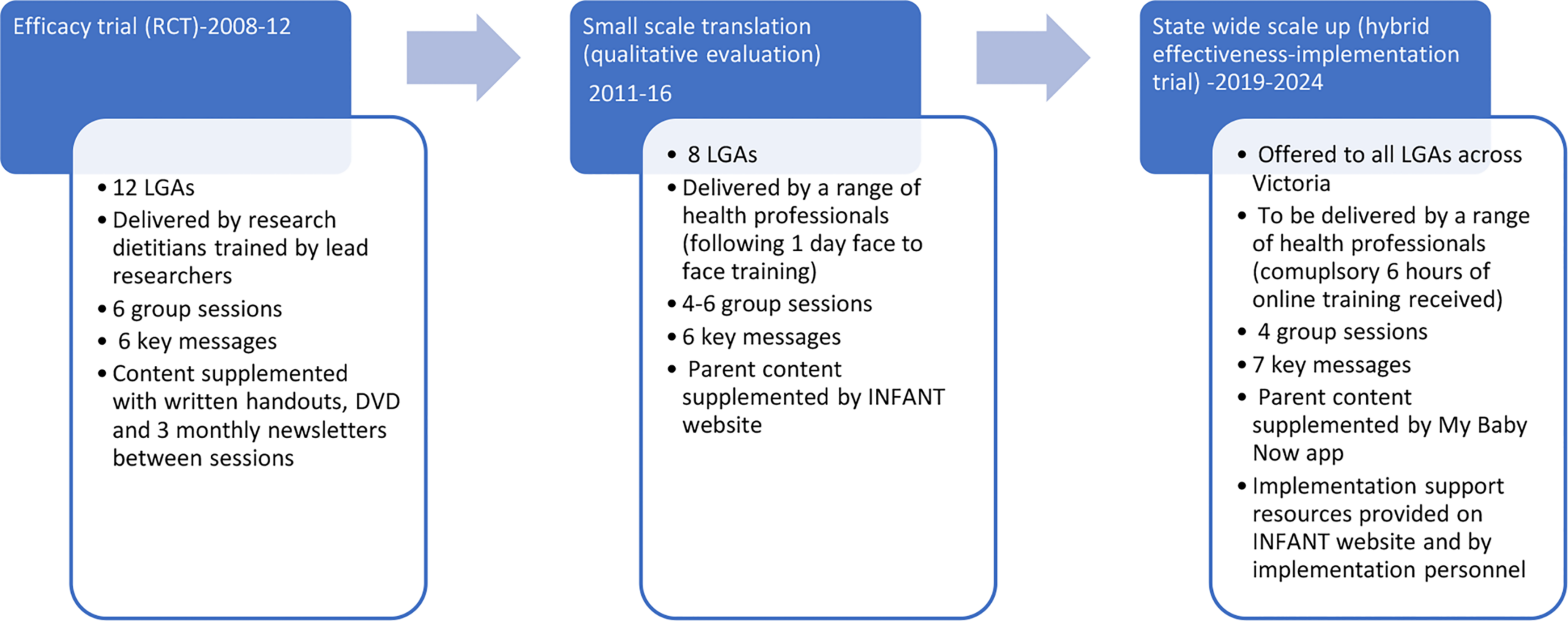
Evolution of INFANT program from efficacy testing to state wide scale up.

**Figure 1** provides an overview of the evolution of INFANT from efficacy testing to state wide scale up. Small scale translation of INFANT across eight Victorian LGAs between 2011-2016 (25) identified a number of barriers to real-world implementation and scale up. These included a high organisational administrative burden coordinating and scheduling group sessions; challenges faced by facilitators in attending face-to-face training; limited staff time and confidence for evaluation (15); parents’ earlier return to work (between 9-12 months as opposed to 15-18 months in the original trial); and parental preference for online supplementary information (15, 26). These findings have informed a number of enhancements to INFANT to improve scalability. These include the provision of online facilitator training, accessible to MCH nurses, dietitians and other health professionals; a reduction in INFANT group sessions from six to four (3, 6, 9, 12 months) to accommodate parents’ earlier return to work; and the development of the My Baby Now app (27, 28). The app provides evidence-based information and support from birth to 18 months of age including two to three age-appropriate push notifications per week to reinforce INFANT key messages (**Box 1**; **Supplementary File 1**). The information from the trial (originally provided *via* handouts and a DVD) has been incorporated into the app, including information from sessions five and six offered at 15 and 18 months in the original trial. Additional information on milk feeding, including a new key message ‘feeding is a learning curve’, was also added to the app and program resources. An overview of the INFANT group sessions and key features of the My Baby Now app are provided in **Supplementary Files 2****,** **3**.

To reflect the move from trial phase to scale up, the acronym INFANT was changed from ‘Infant feeding activity and nutrition trial’ to INfant Feeding, Active play and NuTrition. Health services will be encouraged to offer the app from birth aligned with routine services and through INFANT group sessions starting at 3 months of age. Promotion of the app will be *via* flyers and posters in health services, and SMS and referral during MCH key age and stage consultations. Enrolment of parents to the group sessions will be *via* existing systems for group programs locally. In the original RCT, this involved inviting first time parent groups to continue to meet for INFANT sessions. In the small-scale translation study, areas continued to use this approach, with some making the last session of the first-time parent group (usually when infants were around three months old) the first INFANT session. This opt-in model of enrolment was identified as promoting intervention uptake with parents (26).

Box 1 **INFANT key messages.**

**Table:** 

***Feeding is a learning curve*** Feeding is a learned skill for both parents and babies. It can take time, practice and patience. Breastmilk is all baby needs until around 6months and commercial infant formula is the only safe alternative. Ask a health professional for help to find an approach that works for you.
***Eat together, play together*** From birth, children watch and copy their parents. They learn about their world with you. Enjoy sharing mealtime together and find time for active plav with your child each day.
***Parent provide, kids decide*** Parents provide a range of healthy foods and activities. From these, let kids decide what and how much to eat and do. Keep offering a variety of healthy foods and active play opportunities so they learn to enjoy these with you.
***Snack on veg and fruit*** Eating a wide range of vegetables and fruits is one of the most important things we can do for our health. Vegetables and fruits make great finger foods and are perfect for snacks!
***Colour every meal with veg and fruit*** Try to provide different coloured vegetables and fruits at every meal. It may take up to 10-15 tries before your child learns to like some vegetables, don’t give up! This helps your child to learn to enjoy these foods. Fresh, frozen or canned vegetables and fruits are all great choices.
***Tap into water*** Start to give your baby water in a sippy cup from 6 months of age. From 1 year old, water straight from the tap is the best drink for children. Offer water regularly and make sure that it’s available. Avoid fruit juice, cordial, soft drink, and other sweetened drinks.
***Off and running*** Screens of any type are not recommended at all for children under 2 years of age. Children learn more from you and the world when screens are off. Encourage your child to be active every day and get active together.

### 2.3 Study Design

This study will use a hybrid type II implementation-effectiveness trial design as both implementation and effectiveness outcomes will be studied concurrently (29). Mixed methods will be used to collect data at a system (statewide stakeholders), organisational (health services) and individual (facilitator, parent, infant) level. This study will be conducted over a five year period to allow sufficient time to monitor implementation outcomes with organisations at 12 and 24 months, and for follow up of effectiveness outcomes with children at 18 months of age (**Table 1**).

**Table 1:** Study timeline.

STUDY ACTIVITIES	Yr 1	Yr 2	Yr 3	Yr 4	Yr5
**INTERVENTION UPTAKE**					
Endorsement, dissemination, adoption, implementation, maintenance					
**IMPLEMENTATION TRIAL**					
Reach monitoring & App/website analytics					
Adoption monitoring (6 monthly)					
Implementation survey (pre-training)					
Implementation survey (12 months post training) & qualitative interviews; cost data					
Sustainability survey (24 m post training) & qual interviews					
Implementation data analysis and write up					
**EMBEDDED EFFECTIVENESS TRIAL**					
Comparison arm - recruitment and **T1** baseline data (infants aged <3 m)					
Comparison arm - **T2** post intervention data (infants aged approx.12m)					
Comparison arm - **T3** follow up data (infants aged approx. 18 m)					
Intervention arm - recruitment and **T1** baseline data (infants aged <3m)					
Intervention arm - **T2** post intervention data (infants approx. 12m)					
Intervention arm - **T3** follow up data (infants aged approx. 18 m)					
Analyses & write up – effectiveness measures & economic evaluation					

Two evaluation frameworks underpin the hybrid trial, ‘Outcomes for Implementation Research’ (30) (implementation trial) and ‘RE-AIM’ (31) (effectiveness trial), as outlined in **Table 2**.

**Table 2:** Implementation-effectiveness trial outcomes.

**IMPLEMENTATION**	**Evaluation data**	**Data collection tool**
ORGANISATIONAL READINESS, ACCEPTABILITY, ADOPTION & APPROPRIATENESS (at 6 months)	Number (%) & characteristics - of health services adopting INFANT; - of trained facilitators Facilitator confidence and practices Facilitator/Service level barriers/enablers to adoption/implementation, including organisational readiness	Health Services implementation plan Facilitator training enrolment records Facilitator training pre/post surveys (directly before and after training)
FEASIBILITY & PENETRATION (at 12 months) SUSTAINABILITY (at 24 months)	Number (%) & characteristics - of health services implementing INFANT; - of new/re-trained facilitators Changes in facilitator confidence and practices Facilitator/Service level barriers/enablers to implementation/sustainability Facilitator/Service level adaptation and fidelity	Facilitator training enrolment records Facilitator training follow-up surveys (at 12 and 24 months) Facilitator qualitative interviews Health Services qualitative interviews
COSTS (at 12 months)	Delivery costs	Health Services qualitative interviews
REACH (at 12 and 24 months)	Number & aggregated socio-demographics of participants e.g.: postcode, age, ethnicity, language, education, gender Session and overall completion rates App/website engagement	Health Services records App analytics
**EFFECTIVENESS**	**Evaluation data**	**Data collection tool**
Infant lifestyle pattern score (at 12 and 18 months)	Composite measure of child diet, physical activity and screen time	Online parent surveys (at <3 month, 12, 18 months)
Infant energy-balance behaviours (at 12 and 18 months)	Breastfeeding (at 12 months) Child diet, physical activity, sedentary time	
Infant BMIz (at 12 and 18 months)	Child gender, height, weight	
**COST-EFFECTIVENESS**	**Evaluation data**	**Data analysis**
Implementation Costs	Delivery costs	Costs/outcomes Modelled economic evaluation Cohort multi-state lifetable model
INFANT Effectiveness Outcomes	Infant lifestyle pattern score; Infant energy-balance behaviours; Infant BMIz	

### 2.4 Implementation Trial

#### 2.4.1 Trial Design

INFANT will be implemented in collaboration with eight practice and policy partners including key agencies involved in MCH service delivery, population health and Aboriginal health. Partners will integrate the promotion and endorsement of INFANT to all Victorian LGAs (n=79), leveraging routine communication channels (e.g., statewide newsletters) and via presentations from the research implementation team at existing meetings of service providers. Health services seeking to adopt INFANT will express interest via the INFANT website (infantprogram.org) and be guided through the sign-up process by the research implementation team. Cultural and linguistic adaptations to meet the needs of multi-cultural and Aboriginal and Torres Strait Islander families within Victoria will be explored with relevant peak representative organizations as a sub-project, to be reported elsewhere.

#### 2.4.2 Organisational Eligibility Criteria

To be eligible for adoption, health services will be required to nominate at least one staff member to complete the online INFANT implementation training. Organisational staff eligible to facilitate INFANT include MCH nurses, dietitians, community health workers or nominated personnel. INFANT implementation training comprises eight hours of free online training, providing guidance on all aspects of INFANT implementation and facilitation, with a focus on how to deliver group sessions and integration within existing delivery systems to maximise adoption and sustainability. The training includes case studies of successful implementation, an online discussion forum to promote peer networking (moderated by research staff), and interaction with the research implementation team to develop an implementation plan (a blueprint for local implementation). Following implementation training, health services will be eligible to have their trained staff deliver INFANT with yearly renewal contingent on facilitators completing a short online annual refresher course. Trained facilitators will be provided with online access to all resources including a facilitator and implementation manual as well as parent handouts (downloaded as pdfs) and the My Baby Now app.

#### 2.4.3 Implementation Strategies

Implementation strategies have been selected from the Expert Recommendations for Implementing Change (ERIC) compilation (32) to address barriers identified from the RCT and related small scale implementation studies (15, 26, 28, 33). Key strategies to support local implementation include assessment of organisational readiness; development of an implementation blueprint; implementation support through site visits, resource sharing, and collaborative learning; support with accessing new funding; revising professional roles; implementation audit and feedback; and accessible, incentivised training. **Supplementary File 4** presents the 16 INFANT implementation strategies, categorised according to the six key implementation processes (34): planning, educate, finance, restructure, quality management, and policy context. Implementation strategies are described using Proctor et al.’s (35) recommended ‘specifiying and reporting’ guidelines.

#### 2.4.4 Implementation Outcomes

Implementation outcomes, as detailed below, will be explored to understand (i) the role of, and barriers and enablers to, the use of implementation strategies and perceived impacts on implementation and/or effectiveness, and (ii) the rationale for uptake and modification, and impacts on fidelity and/or effectiveness (30).

##### 2.4.4.1 Organisational Readiness, Acceptability, Adoption, Appropriateness, and Feasibility

Staff who enroll in INFANT implementation training will be invited to complete a quantitative online survey at baseline (pre-training), immediately post-training and at 12 and 24-months post training to assess changes in confidence and practices, to assess barriers and facilitators to implementation, and use of core INFANT messages to inform health professional practice. Facilitators will also be invited to complete a short online survey immediately post-training to provide feedback on the training course. Organisational readiness questions will be embedded in the baseline, 12 and 24-months post training surveys to identify organisational level barriers to adoption, implementation and maintenance. Organisational readiness will be measured using the 12-item ORIC Tool (36) which explores the domains of change valence (motivation), change commitment, change efficacy and change capacity. ORIC scores will be aggregated at organisational (health service) level to inform the development of the implementation plan, tailored to the needs of the organisation. The research implementation team will provide support to organisations to address identified readiness barriers when developing their implementation plans, and during 12- and 24-month qualitative interviews informed by the Consolidated Framework for Implementation Research (CFIR) (37) and the Program Sustainabilty Assessment Tool (PSAT) (38). Number of organisational staff trained and delivering INFANT will be monitored by the research implementation team through six-monthly telephone support. Organisations who have expressed interest or have trained facilitators but do not proceed to implementation will also be invited to participate in qualitative interviews to explore barriers and facilitators to adoption.

##### 2.4.4.2 Fidelity and Adaptation

It is anticipated that contextual modifications will occur, therefore fidelity and adaptation data will be explored using FRAME (39) (Framework for Reporting Adaptations and Modifications-Enhanced) to understand the rationale for modification and impacts on fidelity and/or effectiveness. Questions will be embedded into the 12-months post-training online survey to assess changes to planned INFANT implementation, and the types and context of adaptations made. Qualitative interviews will be conducted with a purposeful sample of these staff (with high/low fidelity, high/low barriers) to explore key factors influencing implementation, and adaptations made to implementation strategies. This will enable the fit of the intervention and implementation strategies to be documented and reviewed (40).

##### 2.4.4.3 Penetration and Sustainability

Factors affecting sustained implementation will be assessed using a quantitative online survey of staff who completed INFANT implementation training 24-months post-training. Qualitative interviews will be conducted with a purposeful sample of organizational staff (continued/discontinued implementation) to explore key factors influencing sustained implementation. Qualitative interviews with key state level partners will also be conducted to explore the extent to which dissemination and promotion was embedded into existing systems, and barriers/facilitators to achieving integration into routine practice.

##### 2.4.4.4 Costs

Costs of scaling up will be examined to assess intervention affordability and sustainability. A mixed-methods approach will evaluate the costs associated with implementation, using qualitative data collected from quarterly semi-structured interviews with key informants involved in implementation and unit costs and quantities by resource category (e.g., time, equipment).

##### 2.4.4.5 Reach

Intervention enrolment, participant characteristics, attendance and completion rates will be monitored over the duration of the project using the MCH nurse record system. Use of the app and website will be measured via analytics using an Engagement Index Score, a novel methodology previously developed by members of the research team (41).

#### 2.4.5 Data Analysis

Descriptive statistics will be reported for quantitative survey items related to organizational readiness, acceptability, appropriateness, feasibility, adaptation and penetration. Qualitative interview data will be transcribed verbatim and coded deductively [informed by CFIR (37), PSAT (38) and FRAME (39)] and inductively to capture issues within and outside of these frameworks. This has been successfully used by the team in previous implementation research (25, 42). Findings will be compared with the choice of INFANT implementation strategies to determine future refinements. Cost data analysis will use a healthcare funder perspective and detailed pathway analysis will specify scale-up and implementation activities in order to measure costs of associated resource use (2023 reference year, costs measured in Australian dollars (AUD)). Total and incremental cost, cost per local government area, cost per participant child and cost per eligible child will be reported.

### 2.5 Effectiveness Trial

#### 2.5.1 Trial Design

An embedded non-randomised controlled trial will assess the effectiveness and cost-effectiveness of INFANT when delivered at scale. The trial will involve comparing primary and secondary effectiveness outcomes between a group of INFANT participants (intervention arm) and a non-randomised comparison group recruited within the same communities prior to the implementation of INFANT (usual care arm). This approach enables comparison of outcomes between parents and infants exposed and not exposed to the intervention living in the same geographical areas and therefore sharing socio-demographic characteristics and having access to the same health services. The risk of bias will be reduced by replicating strategies for recruitment, follow-up and assessment in both trial arms and adjusting for unbalanced baseline characteristics. This trial design was deemed appropriate for assessing the effect of INFANT when implemented at scale in real-world conditions, enabling natural dissemination and uptake to be captured. A cluster randomized design with communities allocated to intervention or usual care was deemed not feasible because the prospect of being randomised to the control arm is likely to reduce participation. Within communities, the prospect of parent groups being randomised to a control arm is unethical (given the known efficacy of the intervention) and contamination between groups would be unavoidable. A stepped-wedge cluster randomised study design (43) was also considered but ruled out as randomisation of LGAs to an implementation time period may interfere with natural uptake under real world conditions.

#### 2.5.2 Eligibility Criteria

Parents will be eligible if they are a first-time parent/primary caregiver of an infant aged 0-3 months, owning an internet enabled personal mobile phone and able to communicate in English. Infants born before 37 weeks gestation will be excluded.

#### 2.5.3 Study Procedures

Families will be recruited over a 6-month period for the comparison arm and over a two-year period for the intervention group (**Table 1**). The comparison (usual care) group will be recruited prior to the implementation of INFANT by health services who have expressed interest prior to undergoing INFANT training. If sample size is not achieved from these LGAs, recruitment will be extended to other LGAs ensuring a mix of metro, regional, rural LGAs and various levels of socioeconomic disadvantage. There will be a three-month gap between recruitment of the last comparison participant and the commencement of recruitment for INFANT implementation participants. This gap ensures comparison participant infants will be too old to enroll in INFANT sessions and thus reduce potential for contamination.

Eligible parents will be invited to participate *via* SMS (with up to 2 reminders) sent by participating organisations. The SMS will contain a link to an online screening and consent process with consenting parents invited to complete online surveys when infants are aged ≤ 3 months (T1), 12 months (T2) and 18 months (T3) (**Table 1**). Up to three reminders will be generated for non-responders to follow up survey invitations. Strategies will be employed to promote retention (e.g. personalised newsletters, reimbursement for time spent completing surveys) which have been used successfully in the original INFANT trial, which achieved 85% retention over two years across both study arms (13).

#### 2.5.4 Primary and Secondary Outcomes

The primary outcome for the effectiveness trial will be child lifestyle pattern score ‘Discretionary food and screen time’ at 18 months. This is a composite measure of child diet, physical activity and screen time derived from a previous analysis (44) of the INFANT RCT (13) and INFANT Extend RCT (33) using principal component analysis. This has been selected as the primary outcome based on previous research (44) confirming the early clustering of these behaviours into unhealthy versus healthy lifestyle patterns in children as young as 18 months of age. Further, these lifestyle patterns track from early childhood into later childhood and adulthood, and are associated with overweight and obesity (45).

Secondary outcomes of the effectiveness trial will include (i) proportion of mothers with any breastfeeding at 12 months; (ii) energy-balance behaviours including child diet (fruit, vegetables, non-core drinks and snacks), child time physically active and sedentary, scores on the ‘Fruit, vegetables and outdoor time’ lifestyle pattern (12 & 18 months), and scores on the ‘Discretionary food and screen time’ lifestyle pattern (12 months) (44); (iii) infant BMIz at 12 months and 18 months, and (iv) child classification as overweight or obese according to WHO definitions (46).

#### 2.5.5 Sample Size

The trial aims to recruit 1,500 primary carer/infant dyads in each arm (total 3,000). Assuming a conservative attrition rate of 33%, it is expected that complete data on 2,000 primary carer/infant dyads (1,000/arm) will be collected. The statistical software PASS version 14.0.9 (NCSS, LLC) was used for all power calculations, assuming that 190 first-time parent groups will run in each arm with an average number of 5.4 children with complete data per group and a coefficient of variability in group size of 0.32, as observed in the INFANT RCT (13) (α=0.05; two-sided tests). A target sample size of 1,000 infants per arm achieves 80% power to detect a mean difference between arms of at least 0.17 points in the lifestyle score ‘Discretionary food and screen time’ at 18 months [primary outcome; standard deviation within clusters (SD) =1.21, intracluster correlation coefficient (ICC) = 0.048]. In our former INFANT trial (44) the difference in “Discretionary food and screen time” pattern score was 0.29 (95% CI: 0.08, 0.49).

The target sample size (1,000 infants per arm) also achieves 80% power to detect the following differences in secondary outcomes:

1) 6.2% increase in the proportion of mothers with any breastfeeding at 12 months assuming the proportion in the control group will be 27% (ICC =0.05);
2) a mean difference (at 18m) between study arms of a) 0.24 serve/day in fruit [SD=1.7, ICC=0.071]; b) 0.25 serve/day in vegetables [SD=2.0, ICC=0.000]; c) 0.06 serve/day in non-core drinks [SD=0.44, ICC=0.034]; d) 0.06 serve/day in non-core sweet [SD=0.39, ICC=0.064]; e) 0.05 serve/day in non-core savoury [SD=0.33, ICC=0.047]; f) 6.7 min/day of screen use [SD=44, ICC=0.11]; and g) 18.4 min/day of physical activity [SD=143, ICC=0.018]. While individual energy-balance behaviour changes appear small, modest increments in energy consumption (126-189kJ/day (30-45 calories)) are estimated to promote population weight gain over time. Collectively these changes have capacity to influence weight gain trajectories at a population level (47, 48);
3) a mean difference between arms of at least 0.13 change in BMIz score by 12 months [SD=1.0, ICC= 0.021]. Assuming a mean BMIz of 0.41, a 0.13 reduction in BMIz to 0.28 equates to a 1.3% reduction in prevalence of overweight and obesity, considered meaningful at a population level.

#### 2.5.6 Data Collection and Analysis

Infant diets will be measured by a validated Food Frequency questionnaire (FFQ) with average percentage categorical agreement against three days of 24 hour recall of 62-64% (49). Infant physical activity will be measured using a single parent-reported question with good test-retest reliability (ICC 0.62) (50). Infant screen time will be measured using a single parent-reported question with excellent reliability (ICC 0.84) (51). Change in BMIz measured at 12 and 18 months will be derived from child height and weight which is routinely collected by MCH nurses and will be made available in de-identified form to the research team. These data are typically available for 80% and 71% of infants at 12 and 18 months respectively (52). Lifestyle pattern scores will be calculated as linear combinations of the standardized dietary (fruit, vegetables, water, discretionary sweet foods, discretionary savory foods), outdoor and television viewing items, using the principal component analysis factor loadings previously published from the combined INFANT and INFANT Extend datasets (44).

Covariates will include self-reported parent socio-demographic characteristics (including marital status, education level, age, sex, Indigenous status, language spoken at home, country of birth, years lived in Australia, health care card holder) parent weight and height, pre-pregnancy weight, self-rated coping, sleep and health, infant sex, birth weight and temperament. Baseline characteristics will be compared between arms using linear mixed models (LMM) or generalized estimating equations (GEE) with link and distribution selected based on the type of variable. The effect of the intervention on the primary outcome (‘Discretionary food and screen time’ lifestyle pattern) will be estimated using a LMM including arm, time (12 and 18 months), interaction arm-time and any baseline variable showing clear imbalance between arms (covariates) as fixed effects. The same LMM model will be used for numerical secondary outcomes. All mixed models will include MCH group as a random effect to account for clustering. The effect of the intervention on proportion of mothers with any breastfeeding at 12 months will be estimated using a GEE model with logit link and binary distribution including arm, time (12 and 18 months), interaction arm-time and baseline confounders. If appropriate, multiple imputation will be used to handle missing data.

#### 2.5.7 Cost Effectiveness

A cost-effectiveness analysis will determine if the implementation of INFANT represents ‘value for money’ when measured incrementally against usual practice. A ‘trial-based evaluation’ (i.e., costs/outcomes exactly as per the trial) will be conducted using primary and secondary outcomes. A ‘modelled economic evaluation’ will also be conducted, using the difference in BMIz between the intervention and control arms, and extending the target population, time horizon and decision context. A cohort multi-state lifetable model (53) will be used to translate changes in infants’ BMIz to long-term health outcomes (healthy adjusted life years). Simulation-modelling using the @RISK software package will calculate 95% uncertainty intervals around epidemiological inputs and cost estimates.

### 2.6 Governance

Practice and policy partners engaged in this proposal are key to the implementation, and importantly the sustained delivery of INFANT within Victorian local government areas. An Implementation Advisory Group consisting of Department of Health representatives (including MCH services), Municipal Association of Victoria representatives (responsible for delivery of MCH services), representatives of local health services who have successfully implemented INFANT for a number of years and the research team meet bi monthly. This group has input into all aspects of this proposal including recruitment and promotion approaches, development and delivery of INFANT implementation training, data collection and study measures. A larger Partnership Advisory committee has also been formed (consisting of all 8 practice and policy partners) meeting annually. This group provides strategic input and advice regarding the alignment of INFANT with organizational and policy directions important for the sustainability of this initiative into the future.

### 2.7 Data Management

All data will be collected and stored electronically in re-identifiable form on Deakin University’s online Research Data Store system. De-identified data will be made available to partners listed in the Partnership Grant and Multi-Institutional Agreement *via* a username and password. Online survey forms include error messages for response outside of plausible values. The de-identified data set may be made available on request to those with a methodologically sound proposal and pending ethics approval.

## 3 Discussion

The evaluation of effective interventions delivered at scale is a crucial, yet under-researched aspect of changing population-level health behaviours and essential to reducing the evidence to practice and policy gap. Examining implementation and effectiveness outcomes concurrently using a hybrid implementation and effectiveness trial design however comes with a number of challenges. Recruitment to the effectiveness trial is contingent on sufficient uptake of INFANT by organisations which in turn is shaped by a wide range of contextual factors outside of the control of the research team. Hence implementation outcomes determine the feasibility of conducting an embedded effectiveness trial within the timeframes of the study. Designing such a trial is also methodologically challenging as traditional trial designs such as RCT, cluster RCTs and stepped-wedge designs, which require organisations to be randomized to control arms, can interfere with the natural uptake of the intervention under ‘real world conditions’. The use of a non-randomized design in this effectiveness trial is therefore not without its limitations but will be minimized by recruiting parents exposed and not exposed to the intervention living in the same communities, replicating strategies for recruitment, follow-up and assessment in both trial arms, and adjusting for unbalanced baseline characteristics.

Successful, sustained implementation is likely due to a combination of factors, including the effectiveness of the intervention and the use of appropriate implementation strategies. An improved understanding of the interrelationship of these factors will therefore enable explicit and transparent decisions regarding intervention adoption, implementation and sustainability. To our knowledge, no obesity prevention interventions in early childhood have been implemented and evaluated at scale under real world conditions. The findings from this study will directly inform future delivery of INFANT in Victoria. It will also provide vital implementation, effectiveness and cost-effectiveness evidence to inform the adoption and sustained implementation of early childhood obesity prevention interventions both in Australia and internationally.

## 4 Ethics And Dissemination

This study has been approved by the Deakin University Human Ethics Research Committee (approval no: 2019-403), and is registered on the Australian New Zealand Clinical Trials Registry (ACTRN12620000670976). Written informed consent will be obtained from all participating implementation staff and parents. In recognition of the importance of research with Aboriginal communities being led by Aboriginal people, Aboriginal families will be the focus of a separate sub-project conducted in partnership with the Victorian Aboriginal Community Controlled Health Organisation and led by Aboriginal researchers. The protocol for this will be published elsewhere and subject to the NHMRC Guidelines for Ethical conduct in research with Aboriginal and Torres Strait Islander Peoples and communities [https://www.nhmrc.gov.au/about-us/resources/ethical-conduct-research-aboriginal-and-torres-strait-islander-peoples-and-communities]. Any future protocol modifications will be submitted for ethics approval and discussed and agreed with the project advisory committee including all chief investigators and partners. While it is unlikely that this health promotion research presents any potential risks associated with participation, if any participant expresses individual concerns or issues related to this study that requires medical attention, the health service will follow their standard procedures. Organisational staff will notify the lead investigator to determine the course of action. Research staff and organisations involved in delivering INFANT will be asked to report any unintended consequences to the research team.

Research findings will be disseminated in peer reviewed journals and at scientific and professional conferences. We will also aim to communicate the study findings at key practice and policy forums and using plain language summaries for participating parents, practice and policy stakeholders distributed *via* email and the program website infantprogram.org.

## Data Availability Statement

The de-identified data set may be made available on request to those with a methodologically sound proposal and pending ethics approval.

## Ethics Statement

The studies involving human participants were reviewed and approved by the Deakin University Human Ethics Research Committee (approval no: 2019-403), and is registered on the Australian New Zealand Clinical Trials Registry (ACTRN12620000670976). The patients/participants provided their written informed consent to participate in this study.

## Author Contributions

All authors contributed to study design which was led by KC, RL with the implementation trial component led by PL, the economic evaluation plan led by VB and MM and the sample size calculation and statistical analysis plan led by LO. RL, PL, and KC developed the first draft of the manuscript. All authors reviewed and commented on the draft manuscript. RL and PL co-wrote the final version of the manuscript. All authors contributed to the article and approved the submitted version.

## Funding

This research is funded by The National Health and Medical Research Council (Grant number: GNT1161223), the Victorian Health Promotion Foundation, and the Victorian Department of Health. KH is supported by an Australian Research Council Future Fellowship (FT130100637). VB is supported by an Alfred Deakin Postdoctoral Research Fellowship. Beyond the peer review process, the funding bodies do not have a role in study design; data management, analysis, nor interpretation; writing of the report; or final authority over the decision to submit study findings for publication.

## Conflict of Interest

The authors declare that the research was conducted in the absence of any commercial or financial relationships that could be construed as a potential conflict of interest.

## Publisher’s Note

All claims expressed in this article are solely those of the authors and do not necessarily represent those of their affiliated organizations, or those of the publisher, the editors and the reviewers. Any product that may be evaluated in this article, or claim that may be made by its manufacturer, is not guaranteed or endorsed by the publisher.
