# Supplementary material for: Protocol for an Effectiveness-Implementation Hybrid Trial to Evaluate Scale up of an Evidence-Based Intervention Addressing Lifestyle Behaviours From the Start of Life: INFANT
Source: Front Endocrinol (Lausanne). 2021 Nov 8;12:717468. doi: 10.3389/fendo.2021.717468 (PMC8715861; doi:10.3389/fendo.2021.717468)
Supplement: Supplementary file 1 [file DataSheet_1.docx]

**Supplementary Table 1: INFANT Key Messages**

| **INFANT Key Message** | **Age-appropriate reinforcement of message at INFANT session** | | | |
| --- | --- | --- | --- | --- |
|  | **3months** | **6months** | **9months** | **12months** |
| ***Feeding is a learning curve***  Feeding is a learned skill for both parents and babies. It can take time, practice and patience. Breastmilk is all baby needs until around 6months and commercial infant formula is the only safe alternative. Ask a health professional for help to find an approach that works for you. | It takes time to get to know your baby’s hunger and fullness signs which should guide when and how much/ how long you feed. | Babies take time to learn to eat solid foods and accept a variety of taste and textures. | Keep offering foods your baby initially rejects, it takes time for them to learn to like many new foods. | Encourage your baby to feed themselves using a range of finger foods and practice with a spoon. |
| ***Eat together, play together***  From birth, children watch and copy their parents. They learn about their world with you. Enjoy sharing mealtime together and find time for active plav with your child each day. | From birth babies watch and copy their parents. | Your baby watches what you eat and drink. Spend lots of time playing on floor together. | Your baby is more likely to want to eat the things they see you eating. | Family meals and playtimes help your baby learn about communication, social interaction and manners. |
| ***Parent provide, kids decide***  Parents provide a range of healthy foods and activities. From these, let kids decide what and how much to eat and do. Keep offering a variety of healthy foods and active play opportunities so they learn to enjoy these with you. | Your job is to respond to your baby hungry signs. Your baby knows how much breast or formula milk to drink. | Provide your baby with a range of healthy food and play options. Let them decide what and how much to eat and do. | Give your baby lots of opportunities to eat healthy foods and be active. Don’t force your baby to eat, they know how much they need. | Fussy eating is normal in toddlers. Keeping offering a wide variety of healthy foods and activities so your toddler can learn to like these. |
| ***Snack on veg and fruit***  Eating a wide range of vegetables and fruits is one of the most important things we can do for our health. Vegetables and fruits make great finger foods and are perfect for snacks! | Have chopped up veg and fruit handy for easy snacks for you. This sets a good example for your baby as they watch what you eat. | When your baby starts solid foods at around 6 months of age, you can offer soft veg first, then fruit (mashed or finger food). | Vege and fruit make great snacks for your baby. Soften hard veg and fruit (like apple, carrot and broccoli) by steaming/microwaving. Once cooled, offer as finger foods. | Even if you think your child won’t eat them – offer veg and fruit as snacks instead of biscuits or other processed food. |
| ***Colour every meal with veg and fruit***  Try to provide different coloured vegetables and fruits at every meal. It may take up to 10-15 tries before your child learns to like some vegetables, don’t give up! This helps your child to learn to enjoy these foods. Fresh, frozen or canned vegetables and fruits are all great choices. | Have plenty of veg and fruit handy for adding to your meals. This sets a good example for your baby as they watch what you eat. | From their first foods at around 6 months of age, babies can enjoy veg and fruit with every meal. | Keep offering a wide variety of veg & fruit, not just those you know your baby likes. They can take up to 15 tries of a food before they learn to like it. | Your baby can now eat most family foods. Adapt your favourite recipes to add more veg to family meals. |
| ***Tap into water***  Start to give your baby water in a sippy cup from 6 months of age. From 1 year old, water straight from the tap is the best drink for children. Offer water regularly and make sure that it’s available. Avoid fruit juice, cordial, soft drink, and other sweetened drinks. | Breastmilk (or infant formula) is the main drink to 12 months of age. Remember busy parents need to drink plenty of water. | Offer boiled water in a sippy cup with meals. Babies don’t need any other drinks apart from breastmilk or formula. | Water should be the main drink for your baby after breastmilk or formula. Fruit juices, cordials and soft drinks are not recommended. Aim to phase out bottles by 12 months of age. | Water straight from the tap is the best drink for kids. You can offer 1-1/2 cups of full fat cows’ milk. All drinks should be offered in cup. |
| ***Off and running***  Screens of any type are not recommended at all for children under 2 years of age. Children learn more from you and the world when screens are off. Encourage your child to be active every day and get active together. | Aim to give your baby 30 minutes of tummy time spread throughout the day. Venture outside for a pram walk and avoid screens which can overstimulate your baby and make it hard for them to sleep. | Babies enjoy the colour, movement and sound of everyday things. Sitting them by a window or watching you cook dinner is more educational than screen. | Set up a safe space for your baby to play and explore. This is much better for their development than offering screens to keep them entertained. | Spend some time playing outside with your child every day. Learning to catch, throw, kick and run takes practise and you are their best teacher. |
