# Supplementary material for: Protocol for an Effectiveness-Implementation Hybrid Trial to Evaluate Scale up of an Evidence-Based Intervention Addressing Lifestyle Behaviours From the Start of Life: INFANT
Source: Front Endocrinol (Lausanne). 2021 Nov 8;12:717468. doi: 10.3389/fendo.2021.717468 (PMC8715861; doi:10.3389/fendo.2021.717468)
Supplement: Supplementary file 2 [file DataSheet_2.docx]

**Supplementary Table 2: Overview of INFANT group sessions content**

| **Content** | **3 months** | **6 months** | **9 months** | **12 months** |
| --- | --- | --- | --- | --- |
| **Introduction** | Parent introductions, ice breaker activity | Recap of last session  Check in on progress/ actions from last session | Recap of last session  Check in on progress/ actions from last session | Recap of last session  Check in on progress/ actions from last session |
| **Feeding** | - Responsive milk feeding (facilitated discussion) - When and how to introduce solids (video) - Signs of readiness for solids and first foods (facilitated discussion) | - Introducing solids-how much, drinks, managing food refusal (video) - Sippy cups, hunger and satiety cues, food textures (facilitated discussion) | - Managing fussy eating and food refusal (video) - Strategies for managing fussy eating, repeat exposure (facilitated discussion) | - Meal time routines (video) - Managing fussy eating in toddlers, meal time routines (facilitated discussion) - Sometimes food and drinks (video) - One thing you would like to do differently with feeding your toddler (facilitated discussion) |
| **Active play** | - Active play at 3 months (video) - Screen time (facilitated discussion) - Tummy time (group activity) | - Active play at 6 months (video) - Baby proofing the home for free play and active play ideas (facilitated discussion) - Active Play (group activity) | - Active play at 9 months (video) - Active play ideas, outdoor play ideas/overcoming barriers (facilitated discussion) - Active Play (group activity) | - Active play at 9 months (video) - Benefits and challenges of free movement for toddlers and strategies for promoting (facilitated discussion) |
| **Healthy happy parents** | Self care and physical activity (facilitated discussion) | Parents own diets and family meals times (facilitated discussion) | Role modelling and ideas for building physical activity into personal and family routines (facilitated discussion) | - Ideas for reducing parents screen time (facilitated discussion)   Sharing of any changes made to parents own diets to role model healthy eating to their toddlers (facilitated discussion) |
| **Wrap up and action setting** | Discussion of key takeaway and one action parents plan to do as a result the session | Discussion of key takeaway and one action parents plan to do as a result the session | Discussion of key takeaway and one action parents plan to do as a result the session | Discussion of key takeaway and one action parents plan to do as a result the session. Options for parents to stay connected |
