# Supplementary material for: Protocol for an Effectiveness-Implementation Hybrid Trial to Evaluate Scale up of an Evidence-Based Intervention Addressing Lifestyle Behaviours From the Start of Life: INFANT
Source: Front Endocrinol (Lausanne). 2021 Nov 8;12:717468. doi: 10.3389/fendo.2021.717468 (PMC8715861; doi:10.3389/fendo.2021.717468)
Supplement: Supplementary file 3 [file DataSheet_3.docx]

**Supplementary Figure 1 – Screen shots of the key features of the My Baby Now app**

| **My baby news feed** – 3 personalised messages per week tailored to babies age and feeding method | **Topic library –** containing 12 topics relating to pregnancy, feeding, recipes, play and parenting. Within each topic evidenced based articles containing both text, videos and images | **Activities-** A range of quizzes and trackers for obtaining personalised feedback | **Facilitated Parent Forum-**for interaction amongst parents, asking questions and reinforcement of key messages |
| --- | --- | --- | --- |
| 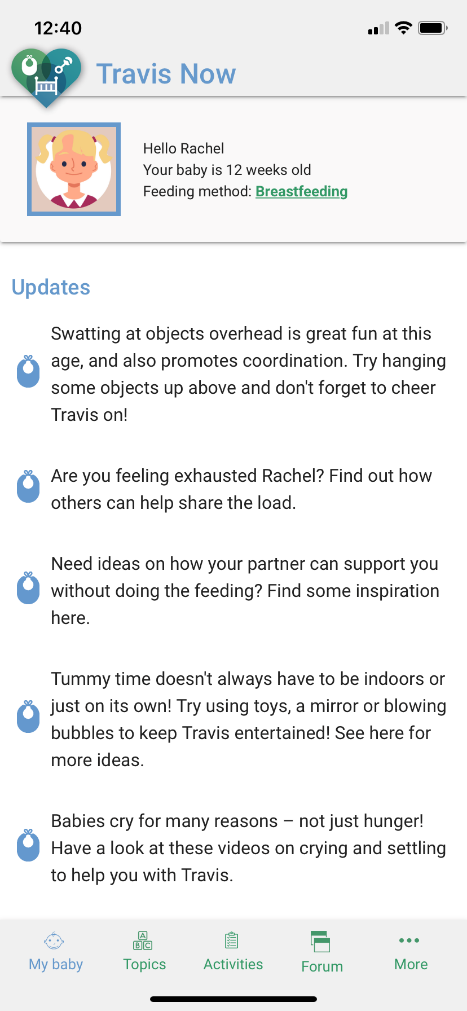 | 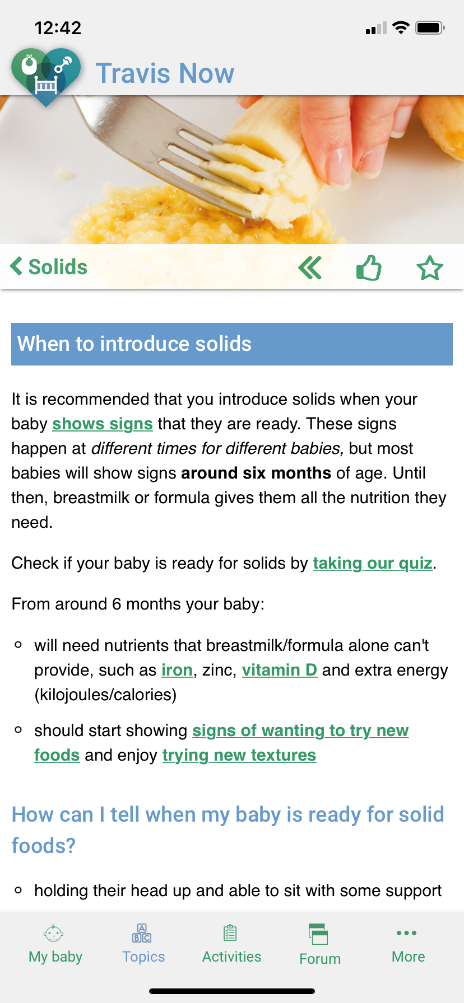 | 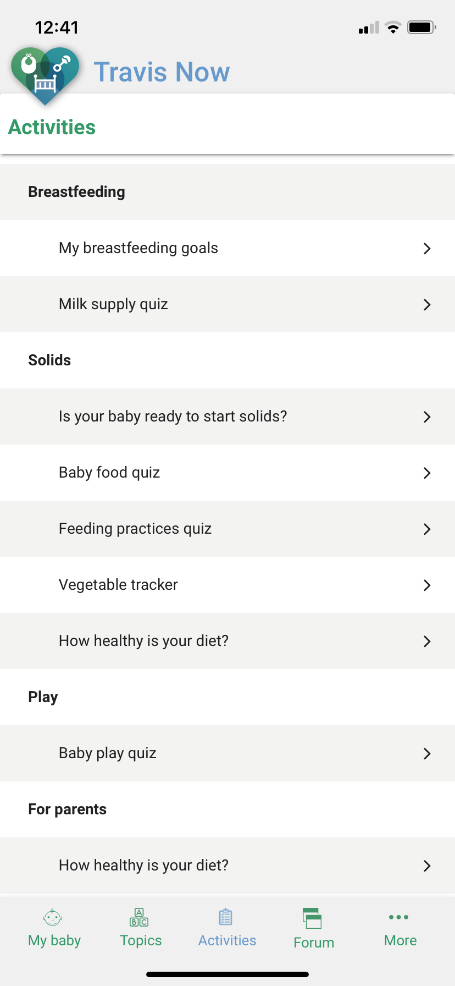 | 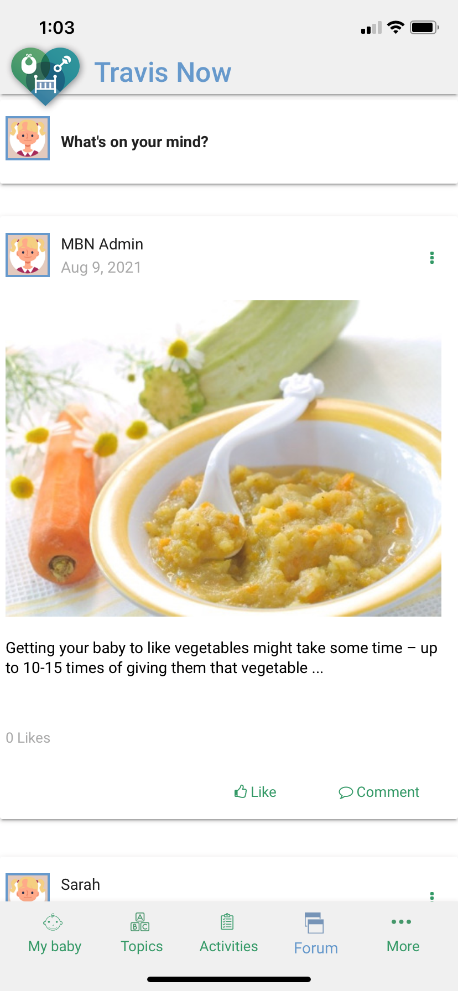 |
