# Supplementary material for: Protocol for an Effectiveness-Implementation Hybrid Trial to Evaluate Scale up of an Evidence-Based Intervention Addressing Lifestyle Behaviours From the Start of Life: INFANT
Source: Front Endocrinol (Lausanne). 2021 Nov 8;12:717468. doi: 10.3389/fendo.2021.717468 (PMC8715861; doi:10.3389/fendo.2021.717468)
Supplement: Supplementary file 4 [file DataSheet_4.docx]

**Supplementary Table 3: Selection of INFANT Implementation Strategies (38)**

| **Category (37)** | **Strategy {**# denotes ERIC implementation strategy} **(34)** | **Actors**  *(deliver strategy)* | **Action**  *(specific action or process)* | **Action target**  *(affected by strategy)* | **Implementation outcome(s)** | **Temporality and Dose** | **Justification** |
| --- | --- | --- | --- | --- | --- | --- | --- |
| PLANNING | #4 Assess for readiness, and identify barriers and enablers | Implementation research team | Scheduled completion of online survey and discussions of organisational readiness | INFANT sites | readiness, adoption, penetration, sustainability | Immediate, 6, 12, 24 months post facilitator training | Assess organization to determine its degree of readiness to implement, barriers that may impede implementation, and strengths that can be used in the implementation effort |
|  | #35 Identify champions AND #36 Identify early adopters | Implementation research team | Scheduled discussions of implementation progress, barriers and enablers | INFANT site facilitators/ implementers | acceptability, adoption, appropriateness, feasibility, penetration | Ongoing post-training | Individuals who will support, promote and drive through implementation, overcoming barriers that may arise |
|  | #63 Tailor implementation strategies |  |  |  |  |  | Tailor implementation strategies to address barriers and leverage enablers identified |
|  | #23 Develop an implementation ‘blueprint’ | Implementation research team | Review and support development of local area INFANT implementation plan | INFANT site facilitators/ implementers | adoption, feasibility, penetration, sustainability | Post-training | Guide implementation progress and strategies through audit and feedback |
| EDUCATE | #16 Conduct educational outreach visits | Implementation research team | In-person or virtual site visits | INFANT sites | acceptability, adoption, appropriateness, feasibility, penetration, sustainability, fidelity, adaptation | 12, 24 months post facilitator training | Provide targeted implementation support to establish and maintain implementation practice |
|  | #29 Develop AND  #31 Distribute  educational materials | Implementation research team; community partners | Develop and disseminate implementation/facilitator manuals, toolkits, and other supporting materials via INFANT website | INFANT site facilitators/ implementers | acceptability, adoption, appropriateness, costs, feasibility, penetration, sustainability, fidelity, adaptation | Ongoing post-training | Provide materials in ways that make it easier for stakeholders to learn about how to implement the intervention |
|  | #19 Conduct ongoing training | Implementation research team; Online training partner | Complete INFANT facilitator training and annual updates; participate in online community of practice | INFANT site facilitators/ implementers | adoption, fidelity | Online training offered 3 times a year; 6-hours initial training; 1-hour annual updates | Enable consistency of knowledge and enhance self-efficacy for implementation of intervention |
|  | #20 Create a learning collaborative | Implementation research team | Establish an online community of practice | INFANT site facilitators/ implementers | penetration, sustainability, fidelity, adaptation | Ongoing post-training | Facilitate a learning community of providers to improve implementation |
| FINANCE | #1 Access new funding | Implementation research team; Funder | Support access to new or existing funding to facilitate local implementation | INFANT sites | acceptability, adoption, costs, feasibility | Post-training | Support time limited actions needed for initial implementation, such as training |
| RESTRUCTURE | #59 Revise professional roles | Organisational level coordination | Scheduled discussions of implementation progress, barriers and enablers | INFANT facilitators | adoption, feasibility, sustainability | 6, 12, 24 months post facilitator training | Revise/expand roles to include implementation of the intervention |
| QUALITY MANAGEMENT | #5 Audit and provide feedback | Implementation research team |  | INFANT site implementers | acceptability, adoption, appropriateness, costs, feasibility, penetration, sustainability, fidelity, adaptation |  | Collect and share progress over a specified time period to monitor, evaluate, and adjust implementation strategies |
|  | #7 Capture and share local knowledge |  |  |  |  |  |  |
|  | #56 Purposely re-examine the implementation |  |  |  |  |  |  |
| POLICY CONTEXT | #22 Create credentialing requirements | Professional Associations –Nursing, Dietetics, Health Promotion | Recognition by professional association of INFANT training as continuing professional education hours | INFANT facilitators | adoption | 6-hours initial training; 1-hour annual updates | Altering continuing education requirements to shape professional practice toward the intervention |

34: Powell BJ, Waltz TJ, Chinman MJ, et al. A refined compilation of implementation strategies: results from the Expert Recommendations for Implementing Change (ERIC) project. Implementation Science 2015;10:21.

37. Powell BJ, McMillen JC, Proctor EK, et al. A Compilation of Strategies for Implementing Clinical Innovations in Health and Mental Health. Medical Care Research and Review. 2012;69(2):123-157.

38. Proctor EK, Powell BJ, McMillen JC. Implementation strategies: recommendations for specifying and reporting. Implementation Science 2013;8:139.
